# MiR-488 inhibits proliferation and cisplatin sensibility in non-small-cell lung cancer (NSCLC) cells by activating the eIF3a-mediated NER signaling pathway

**DOI:** 10.1038/srep40384

**Published:** 2017-01-11

**Authors:** Chao Fang, Yi-Xin Chen, Na-Yiyuan Wu, Ji-Ye Yin, Xiang-Ping Li, Hsuan-Shun Huang, Wei Zhang, Hong-Hao Zhou, Zhao-Qian Liu

**Affiliations:** 1 Departments of Clinical Pharmacology, Xiangya Hospital, Central South University, Changsha 410008, P. R. China; 2 Institute of Clinical Pharmacology, Central South University, Hunan Key Laboratory of Pharmacogenetics, Changsha 410078, P. R. China; 3 Departments of Pharmacy, Xiangya Hospital, Central South University, Changsha 410008, P. R. China; 4 Department of Research, Cervical Cancer Prevention Center, Tzu Chi University, Hualien 970, Taiwan, Republic of China; 5 Hunan Province Cooperation Innovation Center for Molecular Target New Drug Study, Hengyang 421001, P. R. China

## Abstract

Our previous studied indicated that eukaryotic translation initiation factor 3a (eIF3a) increases the sensitive of platinum-based chemotherapy in lung cancer. MiRNAs play an important role in lung carcinogenesis and drug response. In this study, we aimed to identify potential endogenous miRNAs that inhibit eIF3a expression and determine their influence of this inhibition on cisplatin resistance. Using bioinformatics analysis prediction and confirmation with dual-luciferase reporter assays, we found that miRNA-488 inhibited eIF3a expression by directly binding to the 3’UTR of eIF3a. In addition, the overexpression of miRNA-488 inhibited cell migration and invasion in A549 cells, and also inhibited cell proliferation, cell cycle progression by elevated P27 expression. Compared to the parental cell line, A549/cisplatin (DDP) resistant cells exhibited a higher level of miRNA-488. Moreover, we found that miRNA-488 was associated with cisplatin resistance in three NSCLC cells (A549, H1299 and SK-MES-1). The mechanism of miRNA-488 induced cisplatin resistance was that miRNA-488 activated nucleotide excision repair (NER) by increasing the expression of Replication Protein A (RPA) 14 and X*eroderma pigmentosum group C* (XPC). In conclusion, our results demonstrated that miRNA-488 is a tumor suppressor miRNA that acts by targeting eIF3a. Moreover, miRNA-488 also participates in eIF3a mediated cisplatin resistance in NSCLC cells.

Lung cancer, which is characterized by uncontrolled cell growth in lung tissues, is still the most common malignant cancer worldwide^1^,^2^. It can be classified into non-small-cell lung cancer (NSCLC) and small-cell lung cancer (SCLC), and NSCLC counts more than 85% of lung cancer^3^. Platinum-based chemotherapy is the basic therapy in advanced NSCLC^4^,^5^, but the continuous use of these agents often causes chemotherapy resistance in the clinic, which is one of the key factors affecting prognosis^6^. Therefore, a better understanding of the mechanisms of platinum resistance in NSCLC will be important for the development of more reasonable therapeutic approaches for lung cancer treatment.

Micro RNAs (MiRNAs) are small non-coding RNA molecules (containing approximately 22 nucleotides) found in plants, animals, and some viruses. They function in RNA silencing and the post-transcriptional regulation of gene expression by perfectly or imperfectly pairing to the 3’ untranslated region (UTR) of target messenger RNAs (mRNAs)^7^,^8^. Bioinformatics analysis estimated that miRNAs regulate ∼30% of human genes^9^. Notably, miRNA deregulation in cancer could partly result from genomic deletion, mutation, or amplification^10^.

The eukaryotic translation initiation factor 3a (eIF3a) is the largest and core subunit of translation initiation complex 3; it serves as a bridge in the formation of the translation initiation complex and is responsible for ribosomal subunit joining and mRNA recruitment^11^. It is known that eIF3a plays critical roles in the regulation of various gene products, influencing cell growth and proliferation^12^,^13^, differentiation^14^, DNA repair pathways^15^, and cell cycle progression^16^. Recent studies have revealed that eIF3a expression is elevated in several cancer cell lines, while a comparison of the expression levels in human ovary, kidney, lung, breast and colon cancer tissue to normal tissue showed specific high eIF3a expression in lung cancer^17^. Our previous studies found that genotype variation in the eIF3a gene contributes to platinum-based chemotherapy resistance and severe toxicity in lung cancer patients^18^,^19^.

In recent years, ample evidences have revealed that the epigenetic regulation of miRNA alters the pathological progression and prognosis of lung cancer^20^,^21^,^22^. Our latest studies indicated that altered eIF3a expression correlates with the prognosis of non-small lung cancer^23^ and that eIF3a expression was associated with the response of lung cancer patients to platinum-based chemotherapy through the regulation of DNA repair pathways^24^. Based on these works, we sought to further identify the relationship between endogenous miRNAs and the inhibition of eIF3a gene expression. Moreover, we also sought to elucidate how the regulation of eIF3a affects cisplatin resistance in NSCLC. The aim of this study was to provide a new explanation and further understanding of eIF3a action in cisplatin resistance in NSCLC and provide new scientific evidences for eIF3a as a molecular target for personalized pharmacotherapy in NSCLC.

## Results

### A cisplatin sensitive cell line exhibits high eIF3a expression and low miRNA-488 expression, whereas miRNA-488 inhibits eIF3a expression

**Figure 1:**
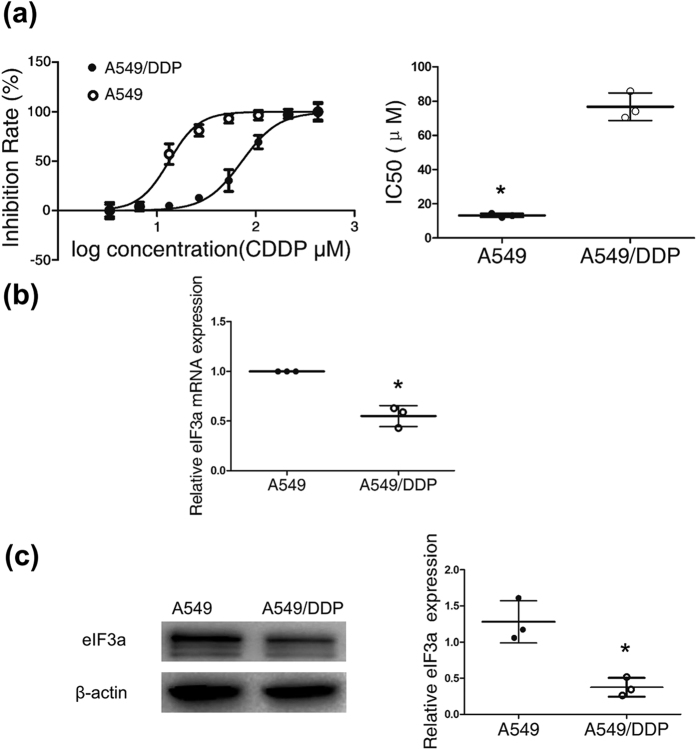
EIF3a showed high expression in a cisplatin sensitive cell line. (**a**) Cells were treated with increasing concentrations of cisplatin (3 μM to 384 μM). Forty-eight hours later, cell viability was tested with MTS. The half maximal inhibitory concentration (IC50) was calculated from 3 independent experiments using GraphPad 5.0 software. The A549/DDP cell line showed a higher IC50 than the A549 cell line. (**b**) The relative expression of eIF3a mRNA in the A549/DDP cell line compared with the parental cell line was measured with qRT-PCR. (**c**) The eIF3a protein expression in the A549/DDP and A549 cell lines was determined by western blot. The bands cropped for the representative images are shown in Fig. 1c, and full-length blots are presented in Supplementary Fig. S3a. EIF3a was highly expressed in the cisplatin sensitive cell line (A549), and the data from three independent experiments is given as the mean ± SD (*P < 0.05).

Firstly, we chose the cisplatin-resistant A549/DDP lung adenocarcinoma cell line and its parental cell line as the research models. The resistance index of A549/DDP was identified by evaluating the half-maximal inhibitory concentration (IC50) value of cisplatin in A549/DDP cells relative to that in the A549 cell line. The IC50 of cisplatin in the A549/DDP cell line was significantly higher than that in the A549 cell line (Fig. 1a).

To confirm that eIF3a is associated with cisplatin chemotherapy resistance in lung cancer, we tested eIF3a mRNA and protein expression in A549 (cisplatin sensitive cell line) and A549/DDP cells (cisplatin resistant cell line). As predicted, eIF3a showed low expression in the A549/DDP cell line and high expression in the A549 cell line at both the mRNA and the protein level (Fig. 1b,c, respectively).

**Figure 2:**
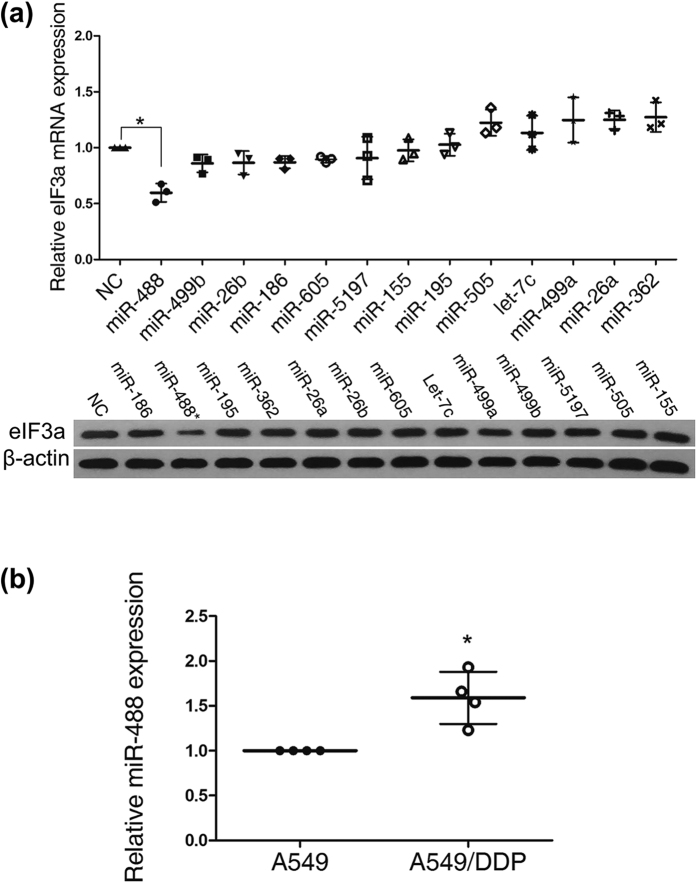
MiR-488 inhibited eIF3a expression and is highly expressed in A549/DDP cells. (**a**) Relative eIF3a mRNA and protein expression levels in the A549 cell line after the transfection of different miR mimics (75 nM). MiR-488 significantly decreased eIF3a mRNA and protein expression. The representative cropping bands are present, and full-length blots are presented in Supplementary Fig. S3b. (**b**) Endogenous miR-488 showed higher expression in the A549/DDP cell line than the A549 cell line. The data from 3–4 independent experiments is given as the mean ± SD (*P < 0.05).

To determine the potential microRNAs regulating the gene expression of eIF3a, the 3’ untranslated region (UTR) of eIF3a was used as a query in the NCBI GenBank database. Subsequently, the potential microRNAs that may regulate eIF3a gene expression were analyzed using TargetScan (http://www.targetscan.org/vert_61), miRBase (http://www.mirbase.org), miRanda (http://www.microrna.org/), miRTarBase (http://mirtarbase.mbc.nctu.edu.tw/index.php) and miRWalk (http://zmf.umm.uni-heidelberg.de/apps/zmf/mirwalk/index.html). Thirteen microRNAs were selected based on these predictions, including miRNA-186, miRNA-488, miRNA-195, miRNA-362, miRNA-26a, miRNA-26b, miRNA-605, let-7c, miRNA-499a, miRNA-499b, miRNA-5197, miRNA-505 and miRNA-155. Then, we tested eIF3a expression in the A549 cell line after the transfection of miRNA mimics. As shown in Fig. 2a, miR-488 significantly inhibited eIF3a expression at both the mRNA level and the protein level.

Showing a negative correlation with eIF3a expression, miR-488 was highly expressed in the A549/DDP cell line and minimally expressed in the A549 cell line (Fig. 2b). These data indicated that miR-488 may be involved in cisplatin resistance in lung cancer by regulating eIF3a expression.

### MiRNA-488 directly targets eIF3a

**Figure 3:**
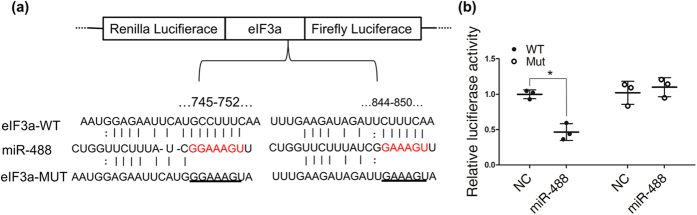
MiRNA-488 directly targeted eIF3a. (**a**) The pmiR-RB-REPORT™ (vector) containing wild-type or mutant eIF3a 3’UTRs was constructed using the matching seed sequence of miR-488 and eIF3a. (**b**) The relative luciferase activity of the indicated eIF3a reporter construct was analyzed in A549 cells. The Renilla luciferase activity of each sample was normalized to the firefly luciferase value and plotted as relative luciferase activity.

The bioinformatics-based target prediction analysis found two binding and interaction regions for miR-488 and eIF3a mRNA (Fig. 3a). Accumulating evidences indicate that miRNAs can inhibit target gene expression through perfect or nearly perfect complementarity to the 3’UTR of the target mRNA. We performed a dual-luciferase reporter assay to detect the potential regulation of eIF3a by miR-488. A miR-488 mimic and a luciferase reporter plasmid containing a wild-type or mutant 3’UTR binding site of human eIF3a were co-transfected into the A549 cell line. MiR-488 significantly inhibited the luciferase activity in A549 cells containing the eIF3a wild-type 3’UTR but failed to suppress this activity in cells with the mutated eIF3a 3’UTR, suggesting that eIF3a is a specific target of miR-488 (Fig. 3b). Combined with the fact that the mRNA and protein expression of eIF3a was suppressed by miRNA-488 over-expression (Fig. 2a), these results indicate that eIF3a is indeed a direct target of miRNA-488.

### MiRNA-488 inhibits A549 cell proliferation, migration and invasion

**Figure 4:**
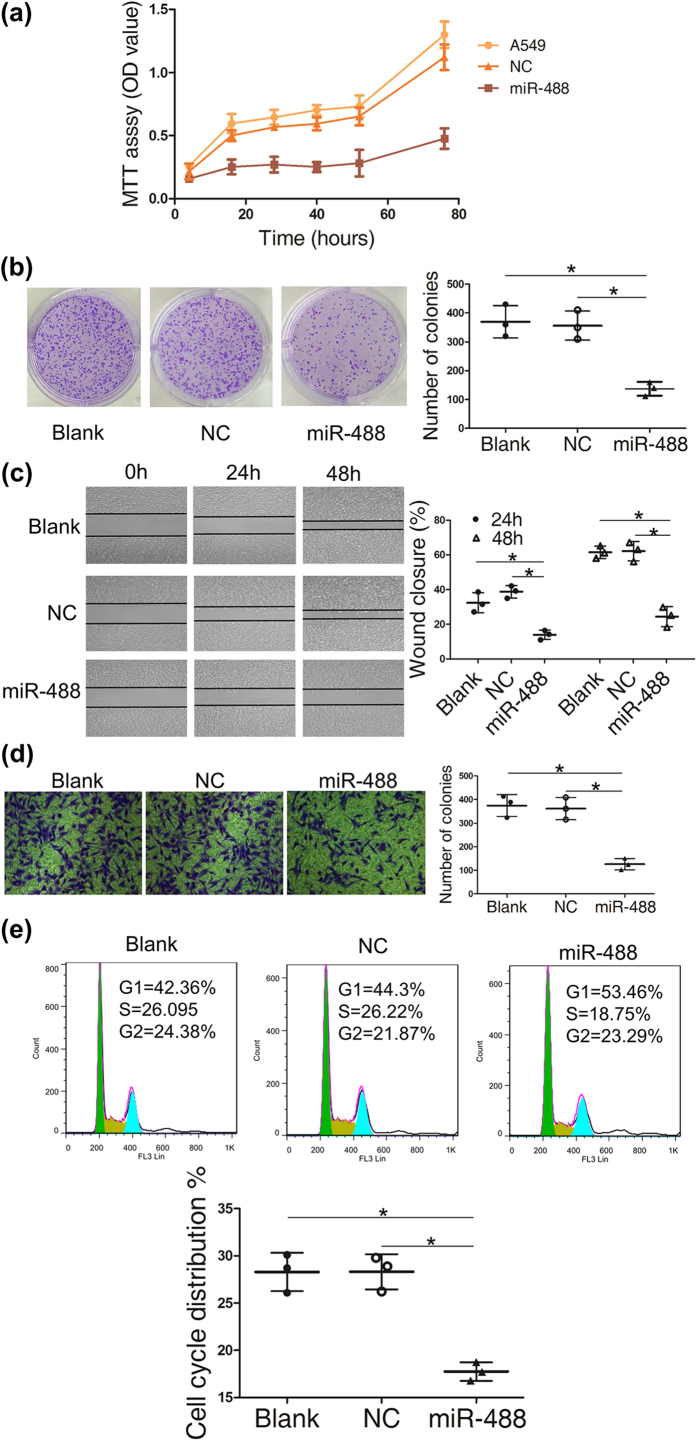
MiR-488 inhibited cell proliferation, migration and invasion in the A549 cell line. The A549 cell line and cells transfected with negative control (NC) and the miR-488 mimic were used in the following assays. Cell viability (**a**) was tested with an MTT assay, and colony formation (**b**) was measured with crystal violet staining. All these showed that miR-488 could inhibit the proliferation of A549 cells. Wound healing (**c**) and transwell assays with Matrigel (**d**) were tested in A549 cells with miR-488 overexpression. The percent of wounds closed or number of cells migrating through the membrane were counted and are compared in the diagrams. The cell cycle (**e**) was evaluated with flow cytometry. The data from three independent experiments are given as the mean ± SD (*P < 0.05).

EIF3a is known as a tumor promoting factor, which induced us to investigate the influence of miRNA-488 on the malignant phenotypes in lung cancer cells. Therefore, we investigated the influence of miR-488 on cell proliferation, migration and invasion by transfecting miR-488 mimics into the A549 cell line. Compared with the negative control and un-transfected group, we found that miR-488 significantly inhibited cell proliferation (Fig. 4a), colony formation (Fig. 4b), cell migration (Fig. 4c) and the invasion (Fig. 4d) ability of the A549 cell line. Cell cycle analysis showed that miRNA-488 reduced the proportion of A549 cells in S phase (Fig. 4e), confirming the result in Fig. 4a that miRNA-488 could slow the rate of cell proliferation.

### MiRNA-488 decrease cisplatin sensitivity in NSCLC cell lines

**Figure 5:**
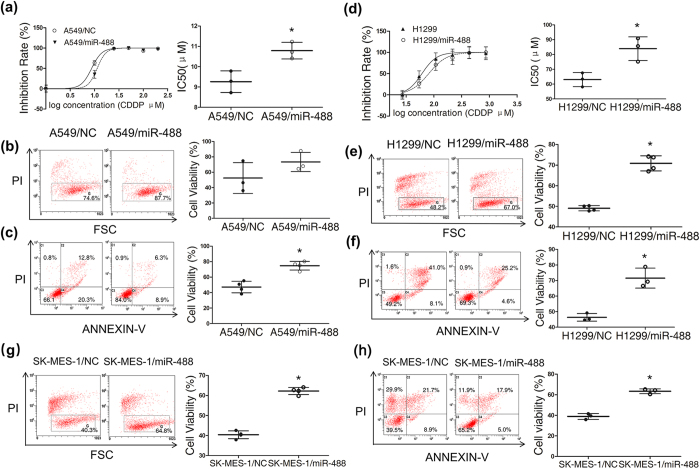
MiR-488 increased cisplatin resistance in three NSCLC cell lines. (**a**) A549 and (**d**) H1299 cells transfected with miR-488 or NC were treated with different concentrations of DDP. Cell viability was tested with an MTS assay. The miR-488 group exhibited a higher IC50 of cisplatin than the NC group. Cell viability was also evaluated using a dye exclusion assay with flow cytometry (**b,e,g**) after the treatment with 10 μM DDP (for A549 cell line) or 60 μM DDP (for H1299 cell line) or 30 μM DDP (for SK-MES-1 cell line). Representative flow cytometry results showing miR-488 effects on cisplatin induced cell apoptosis in A549 (**c**) cells, H1299 (**f**) cells and SK-MES-1 cells (**h**), the right panels of cell viability refer to cells gated in the C3 quadrants. The results of 3–4 independent experiments are given as the mean ± SD (*P < 0.05).

Previous research has shown that eIF3a down-regulation causes cellular resistance to cisplatin in lung cancer^24^, as shown above, we found that miR-488 regulates eIF3a expression via binding to its 3’UTR. Therefore, we evaluated the association between miR-488 expression and cisplatin sensitivity in three NSCLC cell lines. The MTT experiment showed that A549 and H1299 cells were more resistant to cisplatin after miRNA-488 transfection (Fig. 5a,d, respectively); this finding was confirmed with a dye exclusion assay (Fig. 5b,e). A cell apoptosis assay also showed fewer apoptotic cells in the miRNA-488 group compared to the vehicle group after cisplatin treatment (Fig. 5c,f). Additionally, another NSCLC cell line, the SK-MES-1 human lung squamous carcinoma cell also showed the similar results, in which miR-488 transfection increased cisplatin resistance in SK-MES-1 cells detected by dye exclusion and cell apoptosis assay (see Fig. 5g,h). The reduction efficiency of miR-488 to eIF3a in H1299 and SK-MES-1 cells was demonstrated in the supplementary material (see Supplementary Fig. S1a,b, respectively).

### MiRNA-488 increased cisplatin resistance through NER pathway in the A549 cell line

**Figure 6:**
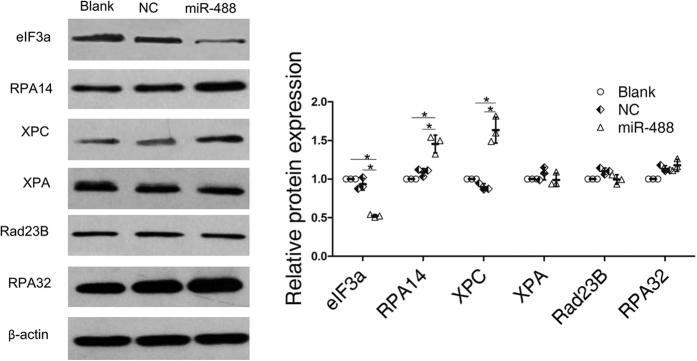
Regulation of NER pathway related proteins by miR-488. Total protein was collected from A549 cells and cells transfected with NC or miR-488. The eIF3a and NER pathway associated proteins XPA, XPC, RPA14, RPA32 and Rad 23B were detected by western blot. The representative cropping bands are present, and full-length blots are presented in Supplementary Fig. S3c. The relative expression levels were determined with ImageJ software. Actin was used as a loading control. The data shown are from three independent experiments (*P < 0.05).

Increased DNA repair is considered one of the major mechanisms of platinum resistance. The DNA damage induced by platinum drugs is primarily repaired by NER^25^. The NER pathway is a complicated multistep process involving multiple proteins, including replication protein A (RPA) and *xeroderma pigmentosum* group proteins such as XPA and XPC^26^. Substantial evidence has suggested that the aberrant expression of these NER proteins was associated with cisplatin resistance^27^,^28^. Although we observed that miRNA-488 inhibited eIF3a expression and decreased cisplatin sensitivity, the mechanism was unknown. In this study, we hypothesized that miR-488 activated NER signaling via directly targeting eIF3a, which increased DNA repair ability and decreased cisplatin sensitivity. Here, we chose A549 cell line as our cell model, western blot showed that the expression of RPA14 and XPC increased after miR-488 transfection (Fig. 6). These results suggested that miRNA-488 may increase RPA14 and XPC expression by targeting eIF3a, resulting in cisplatin resistance.

**Figure 7:**
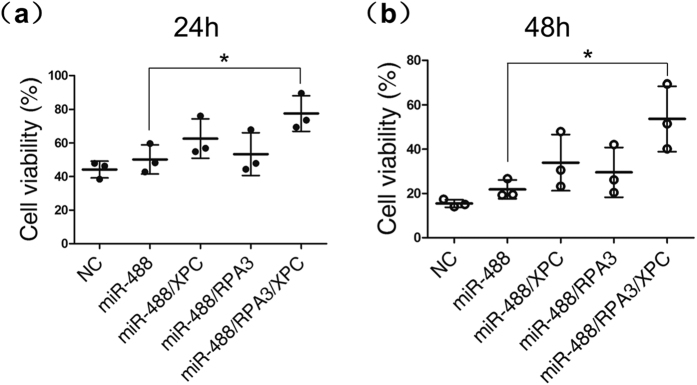
The overexpression of RPA14 and XPC enhanced miR-488 mediated cisplatin resistance. Different combinations of NC, miR-488, expression vector of XPC and RPA14 were transfected in A549 cells, cell viability was tested with an MTS assay at 24 h (**a**) and 48 h (**b**) after treatment with 15 μM DDP. The results of three independent experiments are given as the mean ± SD (*P < 0.05).

We found that A549 cells transfected with miR-488 could elevated the expression of RPA14 and XPC and increased resistance to cisplatin. Further, miR-488 co-transfected with RPA14 or XPC or both in A549 cells all showed variant rise in cisplatin resistance. Notably, the miR-488/RPA14/XPC transfection group had the greatest resistance to cisplatin compared with miR-488 only group (Fig. 7a,b). These results suggested that XPC and RPA14 enhance cisplatin resistance of cells and further confirmed that miR-488-mediating NER pathway in the cisplatin resistance of NSCLC cells. (The protein expressions after transfection were given in the supplement data (see Supplementary Fig. S1c)).

## Discussion

**Figure 8:**
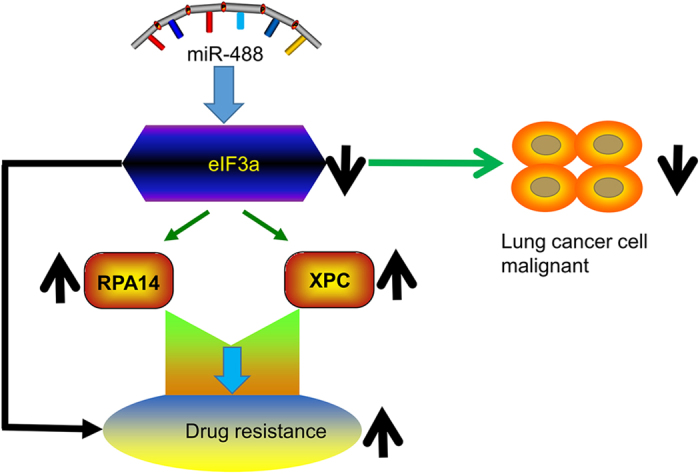
Roles of miR-488 in lung cancer. MiR-488 can directly target eIF3a and down-regulate its expression, which is involved in both the malignant phenotype and the cisplatin resistance of lung cancer.

In this study, we noted that eIF3a was expressed at a lower level in the A549/DDP cell line compared with parental A549 cells, and identified miR-488 as a tumor suppressor that inhibited cell proliferation, migration and invasion in the A549 lung cancer cell line. The mechanism was to lower expression of the eIF3a oncogene. Our study found that miR-488 increased cisplatin resistance in A549 cells, and this result further conformed in other NSCLC cells, H1299 and SK-MES-1. The mechanism of miR-488 induces cisplatin resistance in NSCLC cells is by enhancing NER pathway proteins expression (Fig. 8).

EIF3a was first purified from rabbit reticulocyte lysate^29^ as one of the largest conserved subunits involved in the core functional domain of eIF3, which is the most complex compound involved in messenger RNA translation initiation. Furthermore, eIF3a can interact with many eIF3 subunits, as exemplified by its binding to eIF3b to form a bridge between eIF3b and eIF3c^30^,^31^,^32^. Tian-Rui Xu *et al*. revealed that eIF3a regulated the ERK pathway by binding to Raf-1, and the downregulation of eIF3a resulted in prolonged ERK activation^33^. However, any up-regulators of eIF3a are still unidentified.

It is well known that miRNAs participate in various biological processes, including cell proliferation, cellular differentiation, signal transduction and carcinogenesis^34^,^35^. Herein, based on bio-informatics analysis, we sought to identify the potential miRNA(s) in response to eIF3a regulation. Firstly, by detecting the endogenous eIF3a expression level in A549 cells transfected with different miRNA mimics, we focused on miRNA-488 as a regulator of eIF3a. Then, dual-luciferase reporter assay results showed the reduction of luciferase activity in the miRNA-488 transfected A549 cell line. All these results indicated that eIF3a is targeted by miR-488.

Studies have shown that eIF3a is unregulated in several human cancers, including lung cancer^36^,^37^,^38^, and eIF3a mRNA is also highly expressed in adult proliferating tissues (e.g., bone marrow, thymus and digestive tissues) and in tissues during development (e.g., fetal tissues)^17^. As shown by the evidences above, eIF3a is considered a tumor promoting factor because of its potential role in malignant transformation and the control of cell growth. Moreover, recent studies have found that decreasing eIF3a expression significantly reversed the malignant growth phenotype both in a human lung cancer cell line and in a breast cancer cell line^13^. An increasing number of studies have shown that miR-488 plays critical roles in several diseases^39^,^40^,^41^,^42^. In prostate carcinoma cells, it functions as tumor suppressor by inhibiting cancer cell proliferation and promoting cancer cell apoptosis^39^. Moreover, it suppresses cell migration during chondrogenic differentiation^43^. In the present study, we found that the over expression of miR-488 inhibited cell proliferation, migration and invasion in a lung cancer cell line by directly downregulating eIF3a expression.

Additionally, our lab found that chemotherapy sensitive patients showed higher eIF3a expression in their lung cancers, which was partly ascribed to the role of eIF3a in the regulation of NER pathway proteins^24^. Accordingly, we found lower eIF3a expression in the A549/DDP cell line (a drug resistant lung adenocarcinoma cell line) compared with parental A549 cells. Our results of three NSCLC cells lines suggested that miR-488 increased cell resistance to cisplatin in a mild way, which may be partly due to the increased expression of XPC and RPA14 of the NER pathway when eIF3a is down regulated by miRNA-488.

NER is the most important repair system among several DNA repair pathways in organisms and can prevent gene mutation and repair DNA distortion^44^. RPA is a single-stranded DNA binding protein required for DNA replication and NER; this protein is a heterotrimeric complex consisting of three subunits: RPA1 (RPA70), RPA2 (RPA32) and RPA3 (RPA14), with RPA14 being the smallest subunit^45^. The structure of the RPA-ssDNA complex is essential to DNA damage repair^46^. Salas, T.R. *et al*. provided evidence that RPA3 interacted directly with single-stranded DNA (ssDNA) at the 3′-end of a 31 nt ssDNA molecule^47^. XPC is a conserved DNA repair enzyme involved in the early stages of the global genome repair (GGR) NER pathway with the primary function of DNA damage recognition^48^. Polymorphisms in XPC were reported to be associated with response to platinum-based chemotherapy^49^. Our study showed that RPA14 and XPC levels were elevated after eIF3a was down-regulated by miRNA-488, which was consistent with our previous research in ovarian cancer^50^.

However, we found the miR-488 has inconsistent effect that suppresses eIF3a oncogene expression but increases cisplatin resistant in cells. The chemo-resistant effect by eIF3a inhibition is according to our previous study that cell downregulated eIF3a was resistant to platinum treatment^24^. Further investigation of eIF3a action on cell quiescent are requires to accurate the chemotherapy in NSCLC cells since studies have shown that quiescent cells tend to be quite resistant to all drugs^51^ and our research revealed that A549 cells with miR-488 overexpression would raise more proportion of G1 status but this result could not tell cells between G1 or G0 phase. Studies have shown that maximum expression of P27 is associating to G0 phase cells, implying a role for p27 in maintaining a quiescent state^52^,^53^,^54^. In addition, Dong *et al*. found that the decreased expression of eIF3a could elevate translation of P27^55^, and in our study, we found that the downregulation of eIF3a by miR-488 could increase the expression of P27 (see Supplementary Fig. S2). These may explain why cell proliferation was suppressed and cisplatin resistance was elevated after eIF3a downregulated by miR-488.

Our study has several limitations. First, as the endogenous expression level of miRNA-488 is not high, only the overexpression of miRNA-488 was investigated in the entire study. Second, as one miRNA could target various genes, the direct regulation of the NER pathway proteins by miR-488 was not researched. More studies of miRNA-488 are needed in this area.

In summary, we are first time to revealed that miR-488 directly targets eIF3a and decreases the expression of eIF3a, which play as tumor suppressor to reduce cell proliferation, migration and invasion in a lung cancer cell line. Meanwhile, we confirmed that this regulation functions in cisplatin resistance by increasing the expression of NER pathway proteins, such as XPC and RPA14. The current study provides new clues for NSCLC development and progression under miRNA regulation and novel issues should pay attention in NSCLC treatments.

## Materials and Methods

### Cell culture

The A549 and H1299 human lung adenocarcinoma cell lines, SK-MES-1 human lung squamous carcinoma cell line were purchased from the Chinese Academy of Sciences (Shanghai, China), and the A549/DDP cell line was obtained from the cell biology research laboratory and Modern Analysis Testing Center of Central South University (Changsha, China). All the cell lines were cultured in RPMI 1640 medium (Gibco, Life Technologies) supplemented with 10% fetal bovine serum (FBS, Gibco, Grand Island, NY, USA). Moreover, the A549/DDP cell line was cultured in medium with 2 mg/L cisplatin (Sigma, P4394) to maintain the drug-resistant phenotype before experimentation. All the cell lines were maintained at 37 °C in a humidified atmosphere of 5% CO_2_.

### Cell viability/proliferation and cell cycle and apoptosis assays

Cell viability was tested with the Cell Titer 96^®^ AQueous One Solution Assay (Promega), which was performed according to the instructions after transfection. Cell apoptosis was detected with flow cytometry using an Annexin V FITC Apoptosis Detection kit (BD, USA). The cell cycle was evaluated by flow cytometry with propidium iodide (PI) (Sigma) staining. For dye exclusion assays, cells were analyzed through flow cytometry. PI-positive and -negative cells were considered dead and living cells, respectively. X-axis is Forward-scattered light (FSC) that means proportional to cell-surface area or size.

### Cell clone formation, migration and invasion assays

For clone formation experiments, 1000 cells were seeded into 6-well plates and then cultured for two weeks in medium containing 1% FBS. The cell clusters were stained with crystal violet. The wound healing assay was performed to evaluate cell migration. Cells were cultured for 48 h after being scratched with a pipette tip. For the invasion assay, 2 × 10^4^ cells in serum-free medium were placed into the upper chamber (Costar, Corning, Inc., Corning), which was covered with Matrigel (BD Biosciences, Franklin Lakes). After 48 hours of incubation at 37 °C, the number of cells adhering to the lower membrane of the chambers was counted after staining with a solution containing 0.1% crystal violet and 20% methanol.

### QRT-PCR of miRNA

Total RNA was extracted using a miRNeasy kit (Qiagen), and cDNA was synthesized with a Mir-X™ miRNA First-Strand Synthesis kit (Clontech Laboratories, Inc.). Gene expression was assessed by qRT-PCR using SYBR Premix Dimer Eraser (Perfect Real Time) assay kits. Real-time PCR was performed using the Roche LightCycler 480 PCR System.

### Dual luciferase reporter assay

The recombinant pmir-REPORT Dual-Luciferase vector (Ambion) was used for eIF3a 3’UTR luciferase assays. Cells were cultured in 24-well plates, and wild-type or mutated eIF3a plasmids were co-transfected with miR-488 mimics or NC mimics using Lipofectamine 2000. Forty-eight hours after transfection, firefly and Renilla luciferase activities were measured using the Dual-Luciferase^®^ Reporter Assay System (Promega).

### Western blot analysis

Total protein was collected from cells in RIPA lysis buffer, separated by sodium dodecyl sulfate-polyacrylamide gel electrophoresis (SDS-PAGE) and then transferred onto PVDF membrane (Millipore, Bedford, MA). Then, the membranes were incubated in blocking solution (5% non-fat milk) and probed with primary antibody at 4 °C overnight. The primary antibodies used in this study were as follows: rabbit monoclonal antibodies for eIF3a, P27, Rad23B, RPA32, and XPA (Cell Signaling Technology, USA) and mouse monoclonal antibodies for RPA14 and XPC (Abcam, UK). Beta-actin (Sigma, St. Louis, MO) was used as a loading control.

### Statistical analysis

The statistical analyses were performed using SPSS 18.0 for Windows (IBM, Inc., Chicago, IL, USA). One-way ANOVA was performed for comparison of more than two groups, and the differences between two groups were compared using an Independent Samples T-test. A value of P<0.05 was considered statistically significant. All values were expressed as the mean ± SD.

## Additional Information

**How to cite this article**: Fang, C. *et al*. MiR-488 inhibits proliferation and cisplatin sensibility in non-small-cell lung cancer (NSCLC) cells by activating the eIF3a-mediated NER signaling pathway. *Sci. Rep.* **7**, 40384; doi: 10.1038/srep40384 (2017).

**Publisher's note:** Springer Nature remains neutral with regard to jurisdictional claims in published maps and institutional affiliations.

## Supplementary Material

Supplement Figure
