# Author Correction: MiR-488 inhibits proliferation and cisplatin sensibility in non-small-cell lung cancer (NSCLC) cells by activating the eIF3a-mediated NER signaling pathway

**DOI:** 10.1038/s41598-021-01417-w

**Published:** 2021-11-11

**Authors:** Chao Fang, Yi-Xin Chen, Na-Yiyuan Wu, Ji-Ye Yin, Xiang-Ping Li, Hsuan-Shun Huang, Wei Zhang, Hong-Hao Zhou, Zhao-Qian Liu

**Affiliations:** 1 Departments of Clinical Pharmacology, Xiangya Hospital, Central South University, Changsha, 410008 P. R. China; 2 Institute of Clinical Pharmacology, Central South University, Hunan Key Laboratory of Pharmacogenetics, Changsha, 410078 P. R. China; 3 Departments of Pharmacy, Xiangya Hospital, Central South University, Changsha, 410008 P. R. China; 4 Department of Research, Cervical Cancer Prevention Center, Tzu Chi University, Hualien, 970 Taiwan, Republic of China; 5 Hunan Province Cooperation Innovation Center for Molecular Target New Drug Study, Hengyang, 421001 P. R. China

Correction to: *Scientific Reports* 10.1038/srep40384, published online 11 January 2017

This Article contains an error in Figure 4D, where the image for the blank condition was inadvertently duplicated for the NC panel. The correct Figure 4 and its accompanying legend appear below.

**Figure 4:**
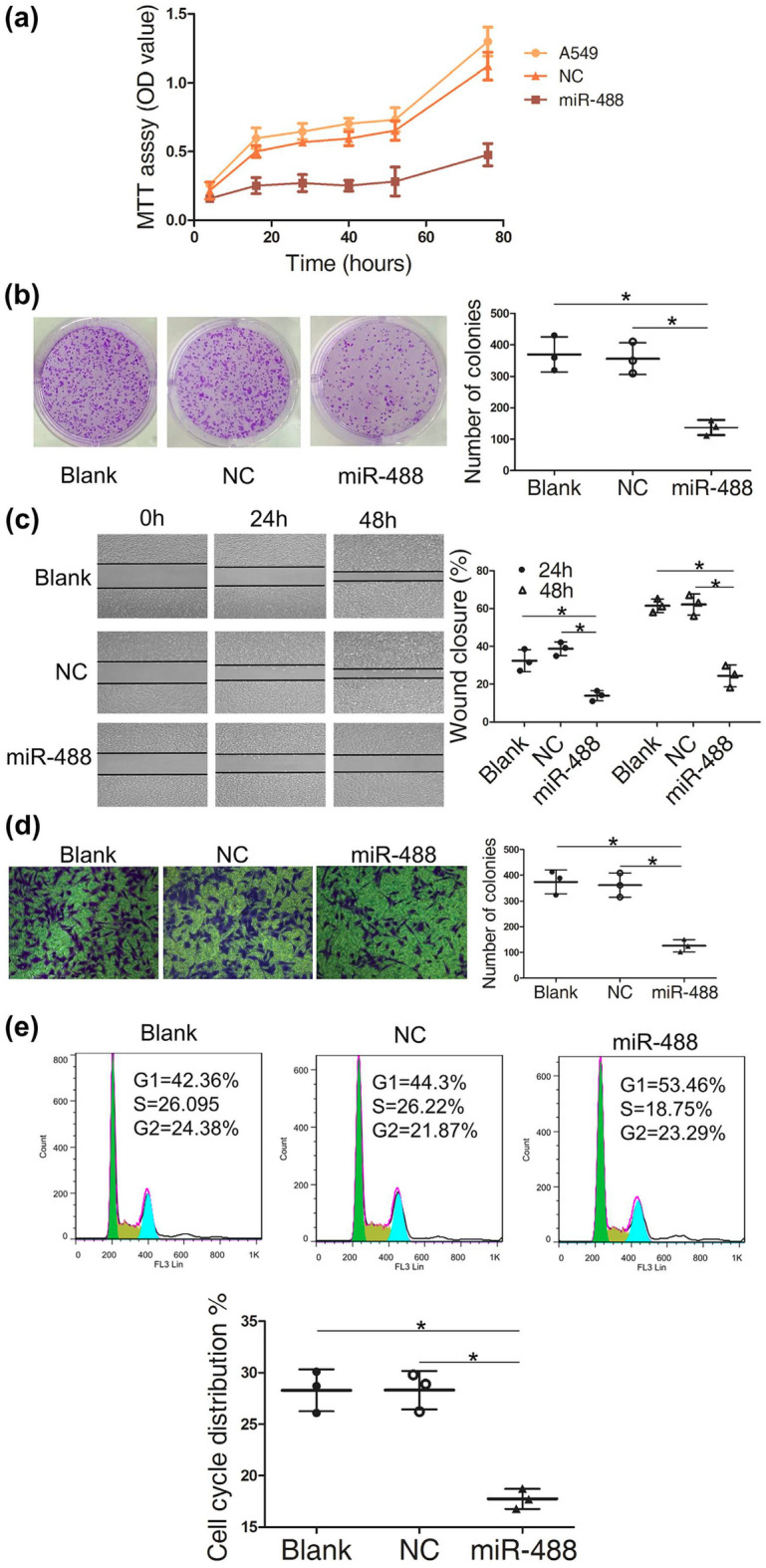
The A549 cell line and cells transfected with negative control (NC) and the miR-488 mimic were used in the following assays. Cell viability (**a**) was tested with an MTT assay, and colony formation (**b**) was measured with crystal violet staining. All these showed that miR-488 could inhibit the proliferation of A549 cells. Wound healing (**c**) and transwell assays with Matrigel (**d**) were tested in A549 cells with miR-488 overexpression. The percent of wounds closed or number of cells migrating through the membrane were counted and are compared in the diagrams. The cell cycle (**e**) was evaluated with flow cytometry. The data from three independent experiments are given as the mean ± SD (*P < 0.05).

