# Supplementary material for: MiR-488 inhibits proliferation and cisplatin sensibility in non-small-cell lung cancer (NSCLC) cells by activating the eIF3a-mediated NER signaling pathway
Source: Sci Rep. 2017 Jan 11;7:40384. doi: 10.1038/srep40384 (PMC5225486; doi:10.1038/srep40384)
Supplement: Supplement Figure [file srep40384-s1.pdf]

MiR-488 inhibits proliferation and cisplatin sensibility in non-small-cell lung cancer (NSCLC) cells by activating the eIF3a-mediated NER signaling pathway

Chao Fang[1,2], Yi-Xin Chen[1,2], Na-Yiyuan Wu[1,2], Ji-Ye Yin[1,2], Xiang-Ping Li[3], Hsuan-Shun Huang[4], Wei Zhang[1,2], Hong-Hao Zhou[1,2,5], Zhao-Qian Liu[1,2,5*]

Departments of Clinical Pharmacology[1] and Pharmacy[3], Xiangya Hospital, Central South University, Changsha 410008, P. R. China; [2]Institute of Clinical Pharmacology, Central South University, Hunan Key Laboratory of Pharmacogenetics, Changsha 410078, P. R. China; [4]Department of Research, Cervical Cancer Prevention Center, Tzu Chi University, Hualien 970, Taiwan, Republic of China; [5]Hunan Province Cooperation Innovation Center for Molecular Target New Drug Study, Hengyang 421001, P. R. China.

* Corresponding author : Professor Zhao-Qian Liu, Department of Clinical Pharmacology, Xiangya Hospital, Central South University, Changsha 410008; P. R. China; Institute of Clinical Pharmacology, Central South University; Hunan Key Laboratory of Pharmacogenetics, Changsha 410078; P. R. China.
Tel: +86 731 84805380, Fax: +86 731 82354476, E-mail: liuzhaoqian63@126.com.

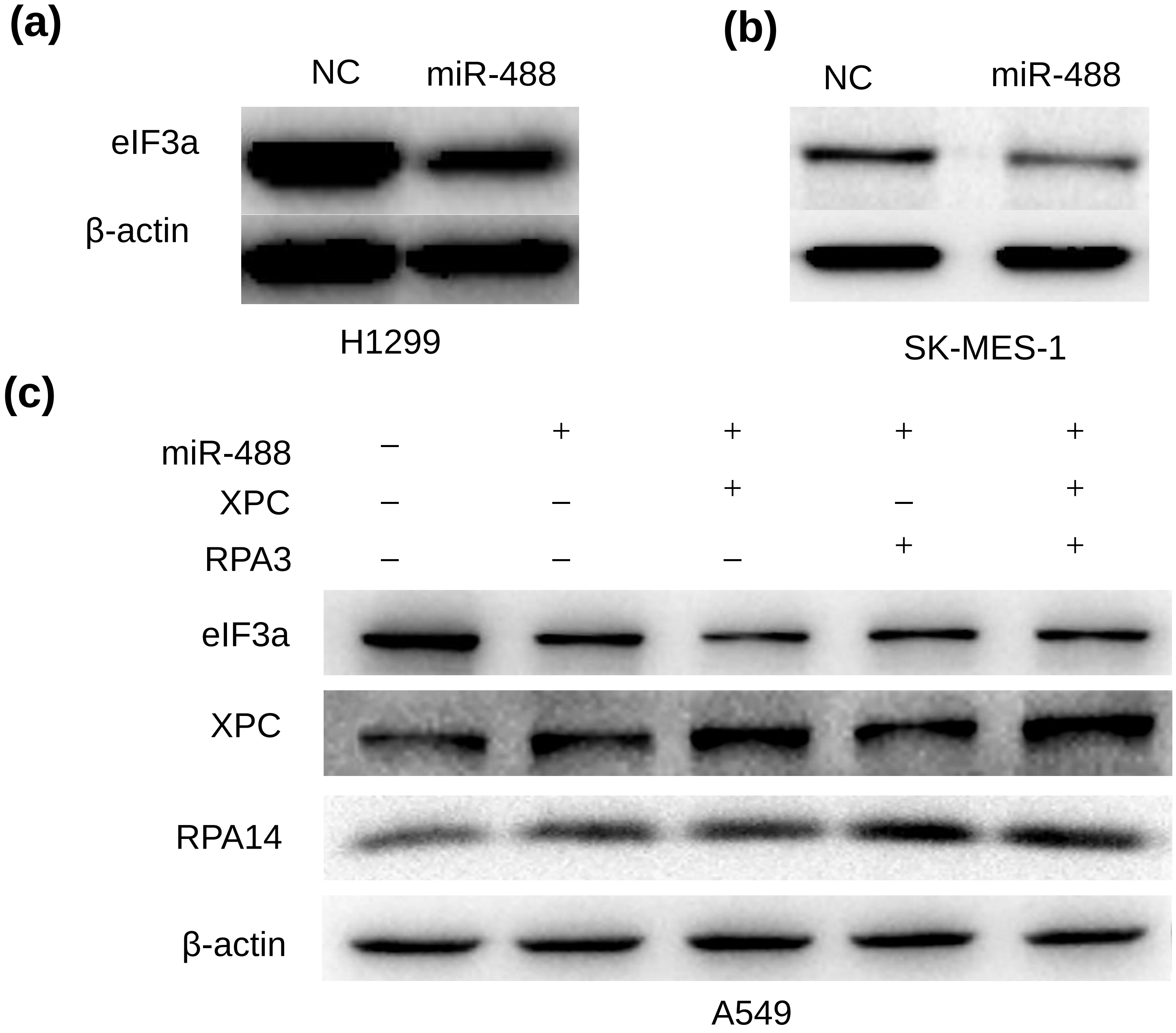

Figure S1. Transfection efficiency of miR-488 on eIF3a down-regulation.

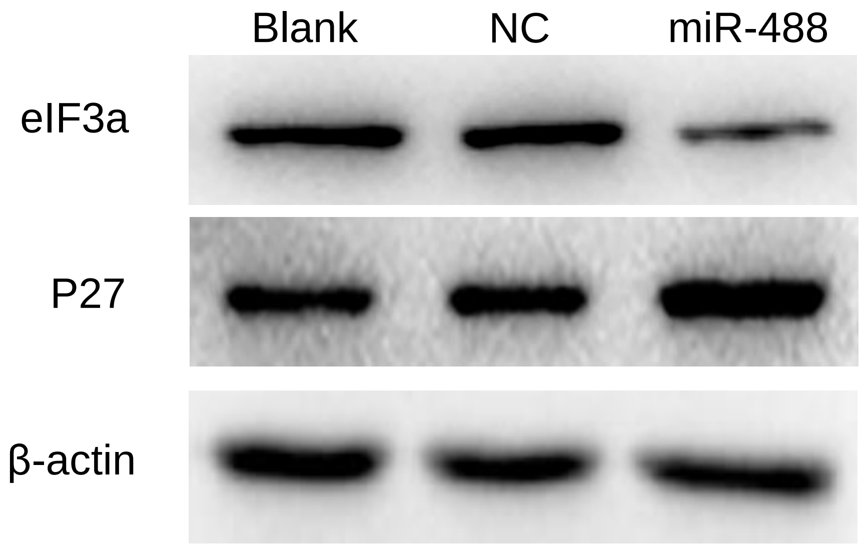

Figure S2. The effect of miR-488 on the expression of P27 protein. Western blot was used to detect the P27 expression after the down-regulation of eIF3a with the miR-488 transfection.

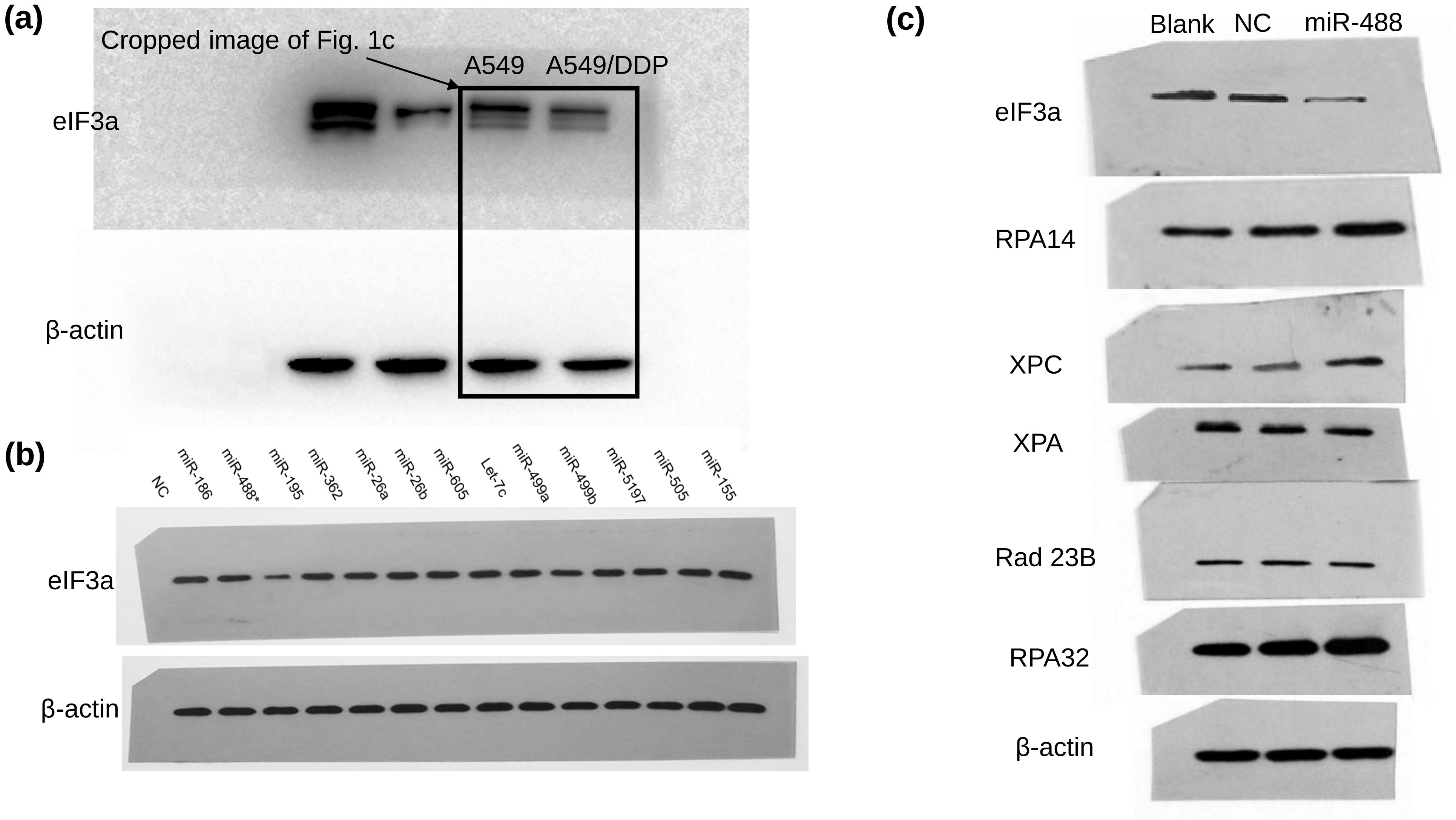

Figure S3. Full-length blots
